# Reclassification of *BRCA1* variants of uncertain significance using ancestry-stratified allele frequencies: A Middle East-focused analysis

**DOI:** 10.17305/bb.2026.14033

**Published:** 2026-06-16

**Authors:** Lama Abujamous, Rozaimi Razali, Hamda Al-Thawadi

**Affiliations:** 1 Department of Basic Medical Sciences, College of Medicine, QU Health, Qatar University, Doha, Qatar; 2 Office of Vice President for Research and Graduate Studies, Qatar University, Doha, Qatar; 3 Department of Biomedical Sciences, College of Health Science, QU Health, Qatar University, Doha, Qatar

**Keywords:** *BRCA1*, variants of uncertain significance, VUS, ACMG/AMP guidelines, ENIGMA *BRCA1/2* specifications, gnomAD, Middle East allele frequencies

## Abstract

Interpreting *BRCA1* variants of uncertain significance (VUS) in underrepresented populations presents significant challenges due to the scarcity of ancestry-matched reference data, which can lead to an overestimation of variant rarity. This study evaluates the effectiveness of employing ancestry-stratified allele frequencies, including a subset from the Middle East, within a calibrated ACMG/AMP framework to enhance the interpretation of *BRCA1* VUS. We analyzed 626 BRCA1 variants from ClinVar, comprising 608 missense, 15 splice-altering, and 3 nonsense variants categorized as VUS or “not provided.” Variants were annotated using gnomAD v4 (both global and ancestry-stratified subsets) and dbNSFP, and subsequently evaluated according to workflows aligned with ACMG/AMP and ClinGen ENIGMA *BRCA1/2* guidelines, with cross-validation performed using the Bayesian point system. Our findings revealed that ten variants (1.6%) were reclassified. Nine missense variants were downgraded to benign or likely benign status based on BS1 and BP1 criteria; notably, none of these downgrades would have occurred if evaluated solely on global gnomAD allele frequencies. Among these, five downgrades relied on the Middle East subset, while four depended on other ancestry-stratified subgroups. Additionally, one splice-altering variant (rs1555582723) was upgraded to likely pathogenic status based on PVS1_Very Strong and PM2_Supporting criteria. In conclusion, utilizing ancestry-aware population data modestly improved the resolution of *BRCA1* VUS, primarily through benign downgrades. This underscores the necessity for larger regional datasets and further functional or case-based evidence to enhance variant interpretation.

## Introduction

Breast cancer is the most prevalent malignancy globally and a primary contributor to cancer-related mortality in women, with an estimated 2 million new cases reported annually [1, 2]. Approximately 5%–10% of these cases are hereditary, typically exhibiting autosomal-dominant inheritance within hereditary breast–ovarian cancer (HBOC) syndromes, driven by pathogenic variants in predisposition genes [3, 4]. *BRCA1* and *BRCA2* are the most extensively characterized high-penetrance genes, accounting for 5%–10% of all breast cancers and approximately 50% of hereditary cases. These genes demonstrate substantial effect sizes (odds ratio [OR] ∼7.6 for *BRCA1*; ∼5.2 for *BRCA2*) and cumulative risks by age 80 of approximately 72% and 69%, respectively [5–8]. Additional susceptibility genes include *ATM, BARD1, CHEK2, PALB2, RAD51C,* and *RAD51D*, with *PALB2* exhibiting risk levels comparable to *BRCA2* in recent analyses [1, 9]. The prevalence and variant spectra of *BRCA1/2* vary by ancestry and geographic region. While most evidence originates from European, Jewish, and East Asian cohorts, African, Mediterranean, and Middle Eastern populations remain understudied, despite emerging data indicating distinct patterns and prevalence estimates across the Middle East and North Africa (MENA) [10–13].

Variants of uncertain significance (VUS) in *BRCA1* represent a significant challenge in hereditary cancer testing, directly impacting genetic counseling, risk communication, and clinical decision-making [1, 7, 14]. *BRCA1/2* VUS are identified in approximately 10%–20% of tests, with higher rates observed in multigene panel and next-generation sequencing analyses [15, 16]. These variants complicate clinical decisions regarding risk-reducing interventions, surveillance, and treatment due to substantial allelic heterogeneity and numerous unresolved missense variants [8, 17–19]. Resolution of these variants necessitates integrated evidence from segregation data, functional assays, computational prediction, and broader representation of diverse populations in genomic research [18, 20, 21].

The underrepresentation of Mediterranean and Middle Eastern populations in *BRCA1/2* reference databases diminishes the accuracy of variant interpretation and genetic risk assessment [22, 23]. The majority of *BRCA* data is derived from European populations, with minimal ancestry-matched information available for MENA groups [24, 25]. Consequently, VUS rates are lower in individuals of European ancestry (approximately 5%–6%) compared to Asian, Middle Eastern, and Hispanic populations (10%–14%), underscoring persistent gaps in population-specific reference data [20, 26].

The Qatar Genome Program is a leading regional initiative addressing these disparities. Phase 1 involved sequencing over 6,000 Qataris, revealing tens of millions of variants, many of which were novel or singletons [20, 27]. Analysis of cancer predisposition genes, including *BRCA1/2*, identified pathogenic variants largely absent from Western datasets and only partially shared with neighboring Gulf and Middle Eastern populations [27]. These findings demonstrate significant heterogeneity in cancer predisposition among Arab groups, with *BRCA1/2* pathogenic variants enriched in some Qatari subgroups, such as those of Persian ancestry, and absent in others, such as those of Arabian Peninsula ancestry [28, 29].

Standardized, auditable rules have substantially reduced VUS uncertainty. The 2015 American College of Medical Genetics and Genomics/Association for Molecular Pathology (ACMG/AMP) framework established a five-tier germline variant classification system comprising 28 weighted criteria and explicit combination rules, enabling consistent interpretation and regular re-evaluation [30–32]. Empirical audits indicate that applying these criteria reclassifies many rare variants and resolves numerous VUS as benign or likely benign. Since 2016, population allele frequencies have become a critical factor in benign classifications, improving inter-laboratory concordance [33–35].

The Bayesian points system quantitatively maps evidence strengths to additive log-odds (Supporting ±1, Moderate ±2, Strong ±4, Very Strong ±8), aligning with ACMG thresholds and facilitating a more transparent resolution of conflicting evidence [36–39]. Gene-specific specifications from the ClinGen Evidence-based Network for the Interpretation of Germline Mutant Alleles (ENIGMA) *BRCA1/2* Variant Curation Expert Panel further operationalize these rules by integrating functional, clinical, and population data, enabling reclassification of a large proportion of *BRCA* VUS with improved reproducibility [31, 40, 41]. This comprehensive framework, incorporating rule-based ACMG/AMP criteria, the Bayesian approach, gene-specific specifications, and ancestry-aware allele frequencies (including Middle East–specific and global datasets), enables transparent and confident classification of many *BRCA1* VUS as benign, likely benign, likely pathogenic, or pathogenic. Although *BRCA1* and *BRCA2* are clinically related, this analysis focuses exclusively on *BRCA1*.

## Materials and methods

### Data sources and initial filtering

All *BRCA1* records were downloaded from ClinVar on August 1, 2025, as a tab-delimited file using the query *“BRCA1[GENE]*”. We retained germline, short variants (<50 bp) restricted to *BRCA1* single-gene entries (≤1 kb). Eligible molecular types included single-nucleotide variants and short indels annotated as missense, nonsense, or splice-related (donor, acceptor, or splice region). Clinical significance was limited to “Uncertain significance” and “Not provided.” To ensure evidentiary consistency, we included only submissions with a review status of “criteria provided, multiple submitters, no conflicts” or “reviewed by expert panel.” The final filtered dataset is provided in Supplementary Data 1.

### Variant call format (VCF) construction

Curated variants were exported from the spreadsheet into an intermediate eight-column text file formatted to match the body of a VCF file. The CHROM field was set to “chr17,” POS was based on the GRCh38 genomic position, ID matched the dbSNP identifier, and REF and ALT were assigned according to the canonical Sequence Position Deletion Insertion (SPDI) representation. The QUAL, FILTER, and INFO fields were set to “.”. A minimal VCF header was added to create list_variants.vcf, which was subsequently bgzip-compressed and tabix-indexed for downstream annotation. The intermediate variant table is available as Supplementary Data 2, while the study VCF and its corresponding index file are provided as Supplementary Data 3a–b.

### Annotation

All annotation procedures were conducted in Ubuntu Linux. The study VCF was annotated with population allele frequencies from Genome Aggregation Database (gnomAD) v4 genomes and exomes, as well as in silico prediction scores from dbNSFP v4.6, including Rare Exome Variant Ensemble Learner (REVEL), Combined Annotation Dependent Depletion (CADD), meta-support vector machine (MetaSVM), meta-logistic regression (MetaLR), meta-recurrent neural network (MetaRNN), Deleterious Annotation of genetic variants using Neural Networks (DANN), Sorting Intolerant From Tolerant for Genomes (SIFT4G), and Functional Analysis Through Hidden Markov Models (FATHMM), using SnpSift. Required resources, such as SnpEff/SnpSift, dbNSFP v4.6, and gnomAD v4 site VCFs with their index files, were downloaded and integrated into a Bash-based workflow. This workflow generated the analysis table by extracting predefined INFO fields from the annotated VCF.

To support downstream interpretation, a simplified dataset containing only fields relevant to this study was created by extracting the necessary information from the fully annotated VCF. These annotation fields were integrated with the assigned ACMG/AMP evidence codes for each variant in a final analytical workbook. This workbook served as the principal dataset for all reported analyses and formed the basis for *BRCA1* VUS reclassification, encompassing both downgrades and upgrades.

The annotated VCF files and their index files are available as Supplementary Data 4a–b (gnomAD v4 annotation) and Supplementary Data 5a–b (dbNSFP v4.6 annotation). A simplified dataset with only study-relevant fields is provided as Supplementary Data 6. These fields were combined with ACMG/AMP evidence codes for each variant in a final analytical workbook (Supplementary Data 7), which served as the main dataset for all analyses and supported *BRCA1* VUS reclassification, including downgrades and upgrades. A fully reproducible workflow, including all commands and configuration files, is available in Supplementary Data 8.

### Evidence assignment strategy (by variant class)

Variant classification was conducted on the annotated VCF using ACMG/AMP criteria within variant-type–specific workflows (Figure 1A–C), adhering to ClinGen ENIGMA *BRCA1/2* Variant Curation Expert Panel (VCEP) specifications. Evidence codes were systematically assessed, with non-applicable codes excluded. Manual review considered gene and transcript context (Ensembl), ClinVar assertions and review status, and selected external resources (e.g., the GWAS Catalog), prioritizing evidence supporting non-VUS classifications.

**Figure 1.**
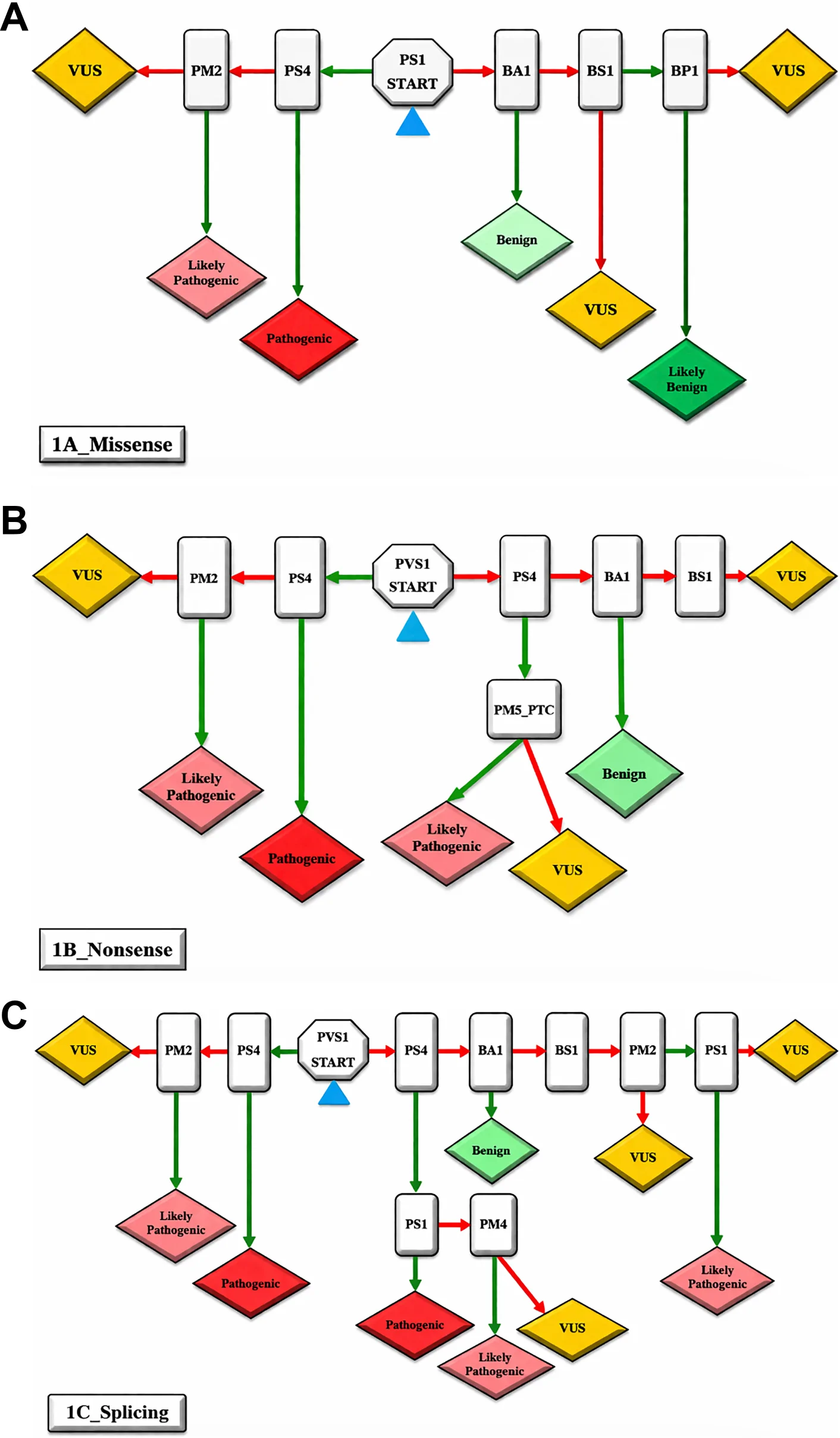
**Workflow for reclassifying BRCA1 VUS using a structured ACMG/AMP approach.** (A) Missense variants were evaluated according to the PS1, BA1, BS1, and BP1 criteria. Both global and ancestry-stratified gnomAD allele frequencies were used to assess BA1 and BS1. Reclassification to benign status required fulfillment of both BS1 and BP1 criteria. PP3 was concurrently reviewed as supportive computational evidence; however, it did not impact the final classification. PS4 was excluded due to a lack of sufficient case-control enrichment data but was retained in the workflow for completeness. (B) Nonsense variants were analyzed using a transcript-aware PVS1 framework, with PM2 applied as supportive evidence when applicable. (C) Splice-region variants were interpreted using PVS1 assignments based on the NM_007294.4 transcript, predicted splice consequences, expected nonsense-mediated decay, and a review of relevant rescue transcripts. Rectangles indicate evidence-assessment or decision steps, whereas diamonds indicate resulting variant classification categories. Green arrows denote pathways leading to reclassification as benign, likely benign, pathogenic, or likely pathogenic. Red arrows denote variants that remain classified as VUS. **Abbreviations**: ACMG/AMP, American College of Medical Genetics and Genomics and Association for Molecular Pathology; BA1, benign stand-alone; BP1, benign supporting; BRCA1, breast cancer 1; BS1, benign strong; gnomAD, Genome Aggregation Database; PM2, pathogenic supporting; PP3, pathogenic supporting; PS1, pathogenic strong; PS4, pathogenic strong; PVS1, pathogenic very strong; VUS, variant of uncertain significance.

**Missense variants (Figure 1A)** were evaluated using a tiered workflow. PS1 was assessed by querying ClinVar for identical amino acid changes, irrespective of nucleotide variation, with at least a two-star germline pathogenic or likely pathogenic assertion. BA1 and BS1 were screened using gnomAD v4 allele frequencies, considering both global and ancestry-stratified subsets, including the Middle East subset (AF_mid). Benign downgrades for missense variants required BS1 in combination with BP1. PP3, defined as CADD scores ≥ 28 and REVEL scores ≥ 0.50, was calculated to support pathogenic evidence for transparency and reproducibility but was not utilized in final variant reclassification for this dataset.

**Nonsense and other loss-of-function variants (Figure 1B)** were evaluated using PVS1, with strength adjusted based on exon position, predicted nonsense-mediated decay, and known rescue transcripts, in line with ENIGMA decision points. PM2 supported rarity but was not employed as standalone pathogenic evidence. Codon-level recurrence consistent with loss of function was treated as an adapted PM5 for protein-truncating variants (PM5_PTC). Variants without a credible loss-of-function mechanism after exon or isoform review were not upgraded by PVS1.

**Splice-altering variants (Figure 1C)** were analyzed. Canonical ±1/±2 splice-site variants were assessed using the *BRCA1* ENIGMA/ClinGen PVS1 decision framework with NM_007294.4 as the primary transcript. PVS1 strength was assigned based on predicted disruption of the canonical splice junction, expected transcript consequences, nonsense-mediated decay competence, and the potential for alternative rescue transcripts or exon context to mitigate loss of function. PM2 was applied at supporting strength only when the variant was absent from both the global gnomAD dataset and the Middle East subset. Non-canonical splice-region variants were not upgraded solely on splicing evidence without stronger functional or transcript data.

### ACMG evidence codes, rules, and scoring

Table 1 outlines the evidence codes (BA1, BS1, PVS1, PS1, PM2, BP1, and PP3) employed in this analysis, detailing their operational rules and corresponding Bayesian point strengths. To ensure an auditable workflow, terminology and strength assignments were standardized across the Methods, Results, figures, and Supplementary Data. Specifically, PP3 consistently indicates supporting pathogenic computational evidence, while PM2 consistently denotes supporting pathogenic rarity evidence.

**Table 1:** ACMG/AMP evidence rules, point strengths, and operational criteria for *BRCA1* VUS

**Evidence code**	**Evidence domain**	**Point score/combining strength**	**Rules**	**Evidence code applied/not applied**
PM2	Population data	1/pathogenic_supporting	Absent from both gnomAD v4 global AF and the Middle East subset (AF_mid). If present in either source, do not award PM2.	Applied > PM2=1 Not applied > PM2=0
BA1	Population data	−8/benign_standalone	Any gnomAD v4 overall or ancestry-stratified allele frequency ≥ 0.001 (0.1%).	Applied > BA1=1 Not applied > BA1=0
BS1	Population data	−4/benign_strong	Any gnomAD v4 overall or ancestry-stratified allele frequency ≥ 0.0001 and < 0.001.	Applied > BS1=1 Not applied > BS1=0
PVS1	Predicted loss of function / splicing	Variable by strength (Very Strong to Supporting)	Applied according to the BRCA1 ENIGMA/ClinGen PVS1 decision framework using NM_007294.4, transcript context, expected splice consequence, nonsense-mediated decay, and rescue-transcript review.	Applied > PVS1 strength recorded Not applied > PVS1=0
PS1	Prior pathogenic missense evidence	4/pathogenic_strong	Same amino-acid change as a ClinVar germline pathogenic/likely pathogenic missense variant with review status ≥ 2 stars.	Applied > PS1=1 Not applied > PS1=0
BP1	Gene/domain-specific benign evidence	−1/benign_supporting	Missense variant occurring outside BRCA1 critical domains (RING aa 2-101, coiled-coil aa 1391-1424, BRCT repeats aa 1650-1857).	Applied > BP1=1 Not applied > BP1=0
PP3	Computational evidence	1/pathogenic_supporting	CADD ≥ 28 and REVEL ≥ 0.50. Evaluated for auditability, but it did not contribute to any final reclassification in this dataset.	Applied > PP3=1 Not applied > PP3=0

Operationalization of ACMG/AMP evidence for *BRCA1* variants of uncertain significance: This table outlines each code (PM2, BA1, BS1, PVS1, PS1, BP1, PP3), detailing its point strength and the applicability rules utilized in our analysis. Population cut-offs were implemented across gnomAD v4 global and ancestry-stratified subsets, including AF_mid (Middle East allele frequency). While PP3 was retained for auditability purposes, it did not influence the final reclassification decision. **Abbreviations**: aa, amino acids; ACMG/AMP, American College of Medical Genetics and Genomics and Association for Molecular Pathology; AF, allele frequency; AF_mid, Middle East allele frequency; BA1, benign standalone; BP1, benign supporting; BRCA1, breast cancer 1; BS1, benign strong; CADD, Combined Annotation Dependent Depletion; ClinGen, Clinical Genome Resource; ENIGMA, Evidence-based Network for the Interpretation of Germline Mutant Alleles; gnomAD, Genome Aggregation Database; PM2, pathogenic supporting; PP3, pathogenic supporting; PS1, pathogenic strong; PVS1, pathogenic very strong; REVEL, Rare Exome Variant Ensemble Learner.

Certain ACMG/AMP codes were not applied due to their specificity to particular variant types or the unavailability of sufficient large-scale data. BS2 (occurrence in healthy adults) was excluded owing to the lack of detailed clinical metadata. PS4 (case enrichment) could not be robustly assessed due to insufficient well-curated case-control or prevalence data for this cohort. BP4 was also excluded because PP3 and BP4 cannot both be simultaneously “Met” for the same variant.

## Results

A comprehensive workbook was developed to integrate all annotations for 626 *BRCA1* variants. dbNSFP provided nearly complete coverage, whereas gnomAD v4 (genomes and exomes) covered approximately 40% of records. This gap in population-frequency data represents a significant limitation for population-based reinterpretation workflows.

### Missense variants

Of the 626 *BRCA1* variants analyzed, 608 (97.1%) were missense. Reclassification adhered to the ACMG/AMP workflow presented in Figure 1A, utilizing PS1, BA1, BS1, and BP1. BA1 and BS1 were assessed against both global and ancestry-stratified gnomAD allele frequencies. No variant met the criteria for PS1 or BA1, nor did any satisfy BS1 using only global gnomAD frequencies. However, by incorporating ancestry-stratified frequencies, 12 missense variants met BS1 in at least one subgroup (Figure 2). Of these, nine were reclassified as benign or likely benign based on BS1 and BP1, while three remained VUS due to insufficient benign evidence (Figure 3). Five of the nine downgraded variants relied on AF_mid for BS1 assignment, and four met BS1 through East Asian or South Asian subgroup frequencies. All nine downgraded variants were originally classified as VUS in ClinVar; the single variant labeled “not provided” was not reclassified. The ENIGMA BRCA1/BRCA2 specifications, ACMG/AMP combining rules, and the Bayesian point-based framework facilitated the accumulation of sufficient benign evidence for these downgrades. PP3 was reviewed as supportive computational evidence but did not influence final classification and is not depicted in Figure 1A. PS4 was not applied due to the lack of robust case-control enrichment data, though it was retained in the workflow for completeness. No missense variant was reclassified as more pathogenic. Table 2 summarizes the 10 reassessed variants, including global allele frequency, AF_mid, subgroup trigger, and classification outcomes under both global-only and ancestry-aware analyses.

**Table 2:** Comparative analysis of the 10 reclassified variants using global-only versus ancestry-aware methodologies

**Variant**	**Global AF**	**AF_mid**	**Trigger**	**Global-only**	**Combined**
c.4337A>G (p.Glu1446Gly) (rs1273755215)	6.8 × 10^−7^	0.0001734	MID	VUS	LB/B
c.4289C>T (p.Pro1430Leu) (rs1597847973)	8.0 × 10^−6^	0	EAS	VUS	LB/B
c.626C>T (p.Pro209Leu) (rs201596327)	8.9 × 10^−6^	0	EAS	VUS	LB/B
c.457A>G (p.Ser153Gly) (rs28897674)	2.7 × 10^−6^	0.0001734	MID	VUS	LB/B
c.659C>G (p.Ser220Cys) (rs431825418)	6.6 × 10^−6^	0	EAS	VUS	LB/B
c.4682C>A (p.Thr1561Asn) (rs56158747)	6.6 × 10^−6^	0	SAS	VUS	LB/B
c.466C>T (p.Leu156Phe) (rs587778115)	4.8 × 10^−6^	0.0004824	MID	VUS	LB/B
c.4288C>G (p.Pro1430Ala) (rs80357466)	2.1 × 10^−6^	0.0001734	MID	VUS	LB/B
c.5574C>G (p.Ile1858Met) (rs876659941)	1.4 × 10^−6^	0.0001734	MID	VUS	LB/B
c.4358-2A>G (rs1555582723)	NA	NA	PVS1_VS + PM2_Sup	VUS	LP

**Abbreviations:** AF, allele frequency; AF_mid, Middle East allele frequency: C;, coding DNA change; EAS, East Asian; LB/B, likely benign/benign; LP, likely pathogenic; MID, Middle East; NA, not available: p., protein change; PM2_Sup, PM2 supporting; PVS1_VS, PVS1 very strong; rs, reference SNP cluster ID; SAS, South Asian; VUS, variant of uncertain significance.

**Figure 2.**
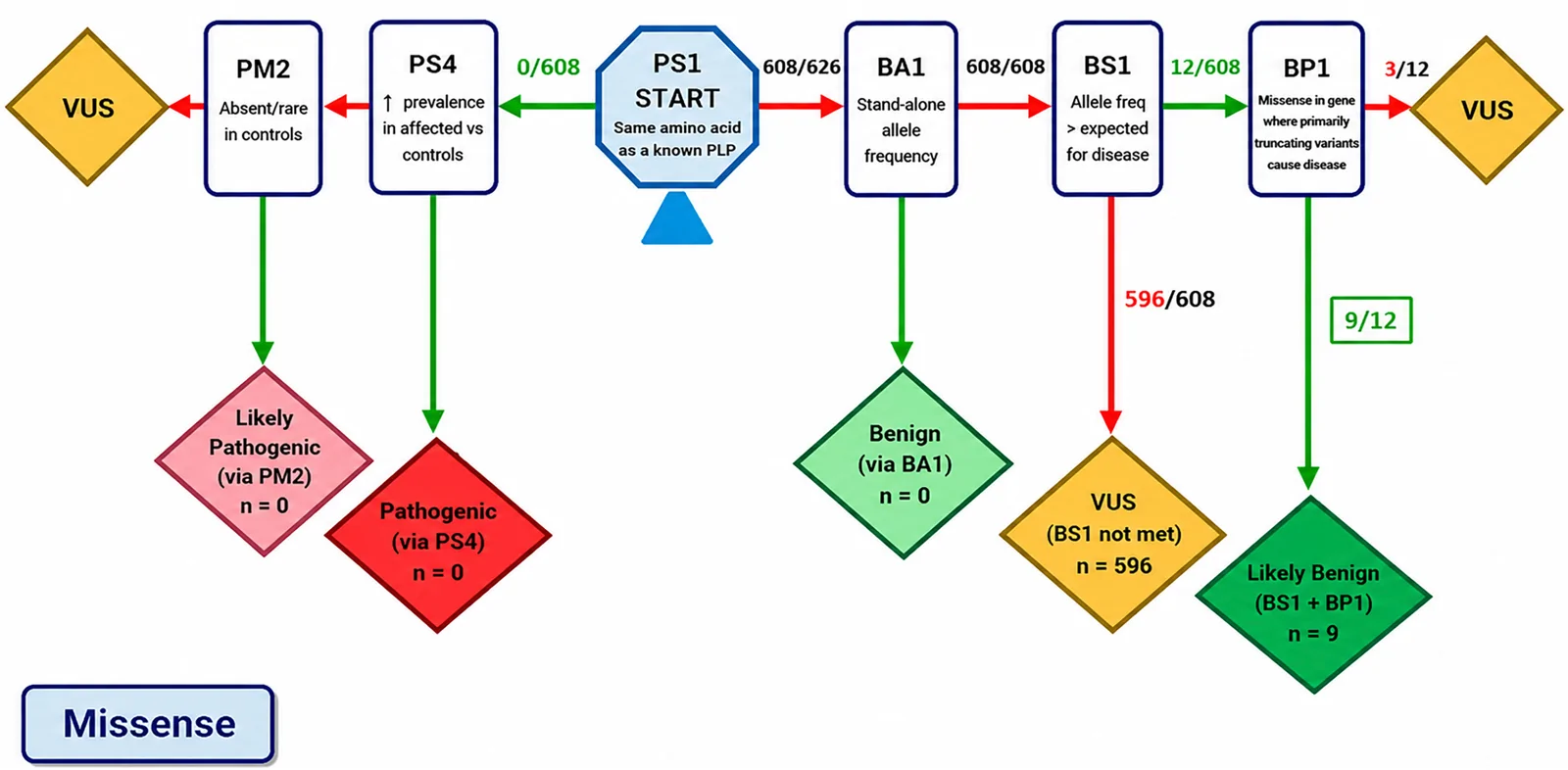
**Workflow and outcomes for the reclassification of missense BRCA1 VUS utilizing a structured ACMG/AMP approach.** Initially, PS1 and BA1 were evaluated; however, no variants met either criterion. Subsequently, BS1 was assessed using both global and ancestry-stratified gnomAD allele frequencies, resulting in 12 of the 608 variants satisfying BS1. Among these, 9 variants were reclassified as benign or likely benign based on BS1 and BP1, while 3 variants remained classified as VUS due to insufficient evidence for reclassification. The remaining 596 variants did not meet BS1 and thus continued to be classified as VUS. PP3 was assessed concurrently, although it is not depicted in the figure and did not contribute to reclassification. PS4 was not applicable to this dataset but was included in the workflow for completeness. The fractions on the arrows indicate the number of variants advancing at each step. Rectangles represent evidence assessment or decision steps. Diamonds represent resulting variant classification categories. Green arrows indicate pathways leading to reclassification as benign, likely benign, pathogenic, or likely pathogenic. Red arrows indicate variants that remain classified as VUS **Abbreviations**: ACMG/AMP, American College of Medical Genetics and Genomics and Association for Molecular Pathology; BA1, benign stand-alone; BP1, benign supporting; BRCA1, breast cancer 1; BS1, benign strong; gnomAD, Genome Aggregation Database; PP3, pathogenic supporting; PS1, pathogenic strong; PS4, pathogenic strong; VUS, variant of uncertain significance.

**Figure 3.**
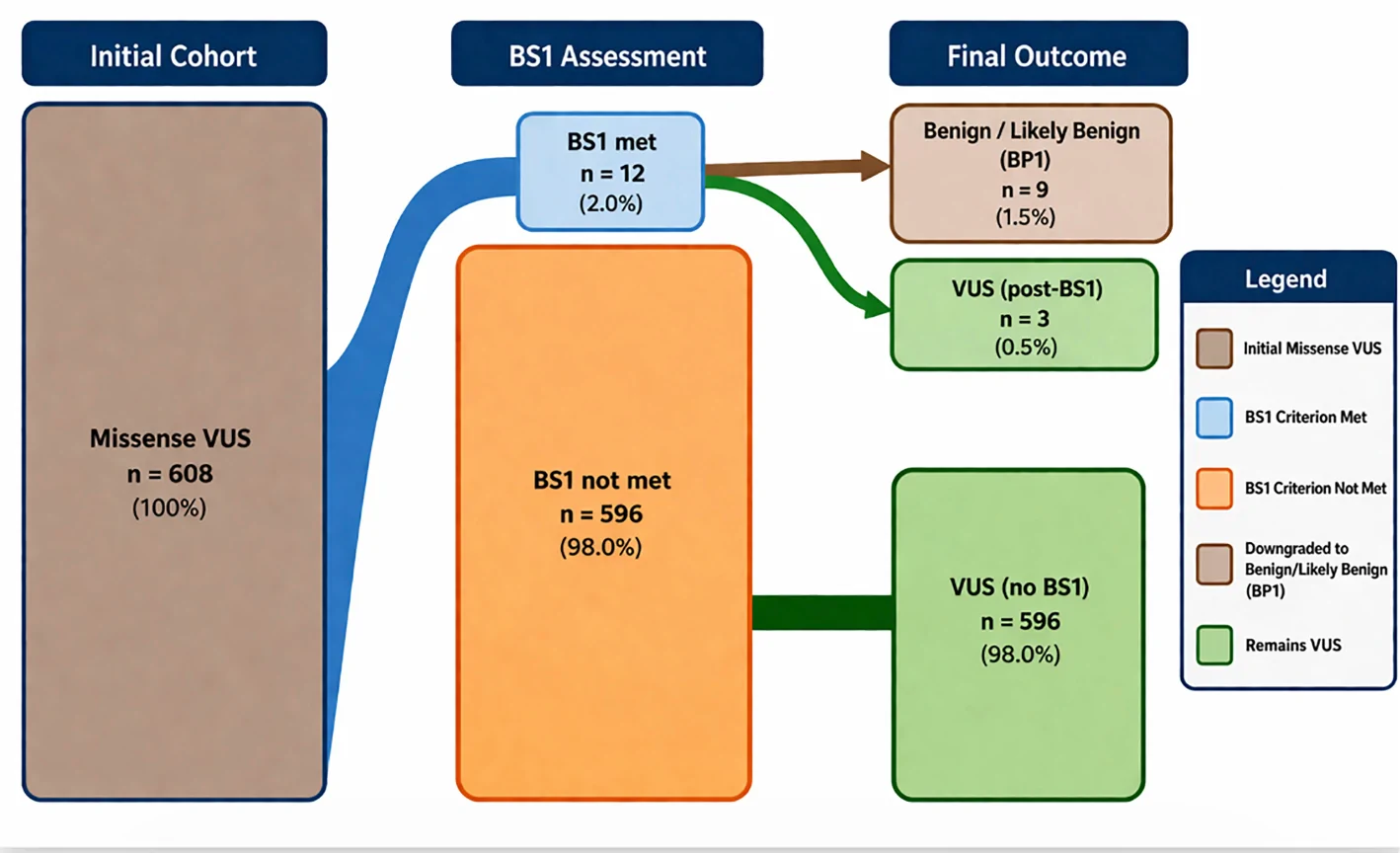
**Sankey diagram illustrating the evaluation and reclassification outcomes of BRCA1 missense VUS through the BS1 criterion.** This diagram represents the flow of 608 BRCA1 missense VUS subjected to BS1 assessment and their final classification. The BS1 evaluation utilized both global and ancestry-stratified gnomAD allele frequencies, identifying 12 variants that fulfilled the BS1 criterion. Of these, 9 variants were reclassified as benign or likely benign in conjunction with BP1, while 3 variants continued to be classified as VUS. The remaining 596 variants did not satisfy the BS1 criterion and thus retained their classification as VUS. **Abbreviations**: BP1, benign supporting; BRCA1, breast cancer 1; BS1, benign strong; gnomAD, Genome Aggregation Database; VUS, variant of uncertain significance.

**Figure 4.**
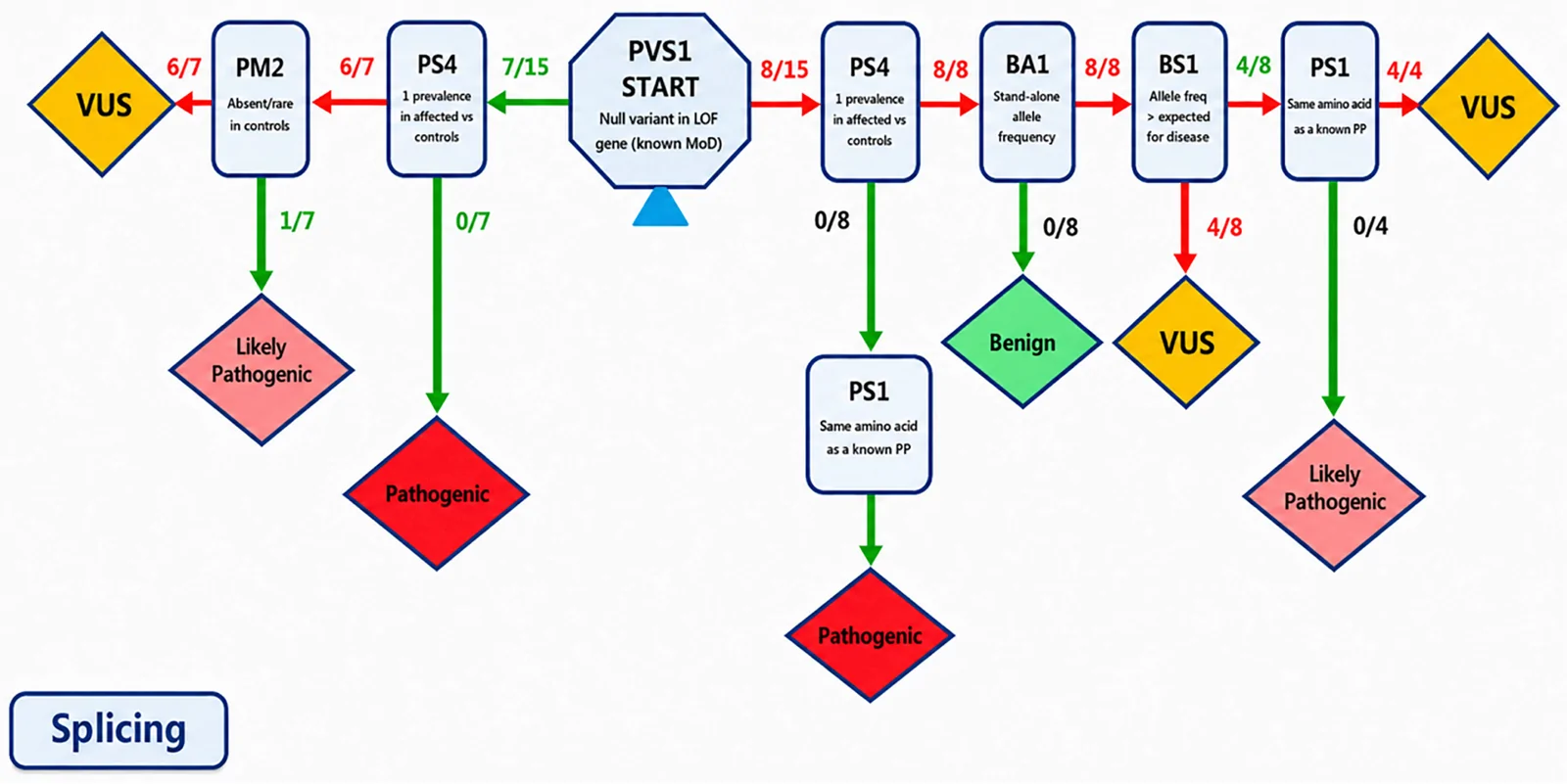
**Workflow and outcomes for the reclassification of splicing BRCA1 VUS.** The splicing BRCA1 VUS were assessed using a PVS1-focused ACMG/AMP workflow in accordance with ENIGMA guidelines. The strength of PVS1 was evaluated based on predicted splice disruption, transcript context, and anticipated nonsense-mediated decay. Of the 15 splice-altering variants analyzed, seven received a non-zero PVS1 assignment, while the remaining eight proceeded through the alternative downstream evaluation pathway. One variant met PVS1_Very Strong together with PM2_Supporting and was reclassified as likely pathogenic; the remaining variants retained their classification as VUS. The remaining variants did not provide sufficient evidence for reclassification and were retained as VUS after a comprehensive review of the pertinent criteria. Rectangles represent evidence-assessment or decision steps. Diamonds represent resulting variant classification categories. Green arrows indicate pathways leading to reclassification as benign, likely benign, pathogenic, or likely pathogenic. Red arrows indicate pathways in which variants remain classified as VUS. **Abbreviations**: ACMG/AMP, American College of Medical Genetics and Genomics and Association for Molecular Pathology; BRCA1, breast cancer 1; ENIGMA, Evidence-based Network for the Interpretation of Germline Mutant Alleles; PM2, pathogenic supporting; PVS1, pathogenic very strong; VUS, variant of uncertain significance.

**Figure 5.**
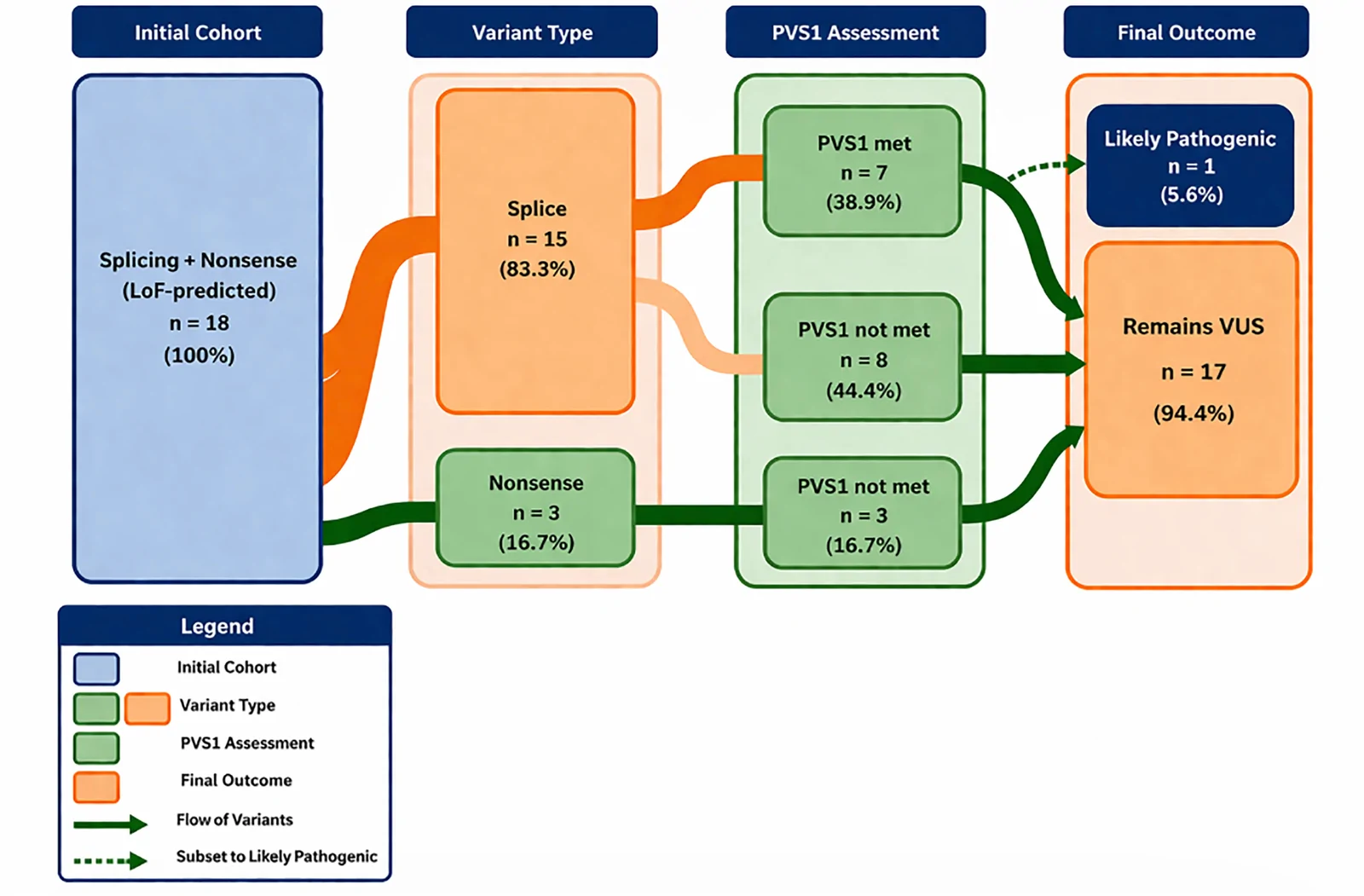
**Sankey diagram of reclassification of splice-altering and nonsense BRCA1 variants.** Among the 18 BRCA1 variants predicted to result in loss of function, 15 were classified as splice-altering and 3 as nonsense. Seven splice-altering variants received a non-zero PVS1 assignment. One variant was reclassified as likely pathogenic based on PVS1_Very Strong and PM2_Supporting criteria. The remaining 17 variants, comprising 14 splice-altering and 3 nonsense variants, retained their classification as VUS. **Abbreviations**: BRCA1, breast cancer 1; PM2_Supporting, PM2 supporting; PVS1, pathogenic very strong; PVS1_Very Strong, PVS1 very strong; VUS, variant of uncertain significance.

### Splice-altering and nonsense variants

Among the 626 variants, 15 (2.4%) were splice-altering and 3 (0.5%) were nonsense, totaling 18 predicted loss-of-function candidates. Seven splice-altering variants exhibited a non-zero PVS1 strength following transcript-level evaluation (Figure 4). Only rs1555582723 (NM_007294.4:c.4358-2A>G), which affects the canonical -2 splice acceptor site, was reclassified as a loss-of-function event compatible with a transcript subject to nonsense-mediated decay. Due to insufficient rescue transcript evidence, this variant met PVS1_Very Strong and, combined with PM2_Supporting, was classified as likely pathogenic. The remaining splice-altering and nonsense variants were classified as VUS due to lower PVS1 strength, absence of PM2, or a transcript context that did not support a stronger loss-of-function interpretation (Figure 5).

These classifications were verified using the ACMG/AMP point-based framework and combination rules. Under the strength-aware ENIGMA/ACMG interpretation, rs1555582723 (PVS1_Very Strong plus PM2_Supporting) was classified as likely pathogenic. Variants rs1567788925 and rs80358178 (PVS1_Moderate with or without PM2_Supporting), as well as rs1353755235, rs397509150, rs80358155, and rs876658790 (PVS1_Supporting with or without PM2_Supporting), remained classified as VUS. The point totals consistently supported these classifications. This comparison underscores the critical importance of maintaining evidence strength, as collapsing all PVS1 or PM2 observations into unweighted ACMG labels would have inappropriately overstated pathogenicity for several splice-altering candidates.

## Discussion

### Summary of key findings

We developed a transparent, variant-type-specific framework to re-evaluate *BRCA1* VUS from ClinVar. This framework integrated ACMG/AMP criteria, ClinGen ENIGMA gene-specific specifications, and a complementary points-based system, with population-frequency evidence serving as the primary factor. Although only 10 of 626 variants (1.6%) were reclassified, a direct comparison revealed that ancestry-stratified data provided additional value beyond global gnomAD data. Global frequencies alone did not result in any missense variant downgrades, whereas ancestry-specific subsets enabled nine benign downgrades, five of which relied on the Middle East subset.

### Role of population allele frequencies (including Middle East subsets)

Population allele frequencies were the primary driver of benign reclassification, indicating that this study represents a population-calibrated reinterpretation analysis rather than a comprehensive evaluation of all ACMG/AMP evidence types [42]. The Middle East subset provided incremental, albeit modest, value for certain variants. Of the nine downgraded missense variants, five required AF_mid to meet BS1, while four were already supported by other ancestry-stratified gnomAD subgroups. These results support the utility of ancestry-aware reference data and recommend careful interpretation of region-specific effects.

To mitigate false rarity, PM2 was assigned only at supporting strength and exclusively when a variant was absent from both the global gnomAD dataset and the Middle East subset. This conservative approach prevents over-weighting rarity in underrepresented populations but also highlights a limitation: gnomAD coverage was incomplete for many study variants, and the Middle East subset is relatively small. Larger regional reference panels and local in-house frequency datasets are expected to further improve calibration.

Systematic misclassification due to under-representation of certain ancestries is well documented. Many ClinVar pathogenic/likely pathogenic (P/LP) assertions are downgraded when appropriate allele frequency data are used, and only a small proportion of variants with an allele frequency greater than 5% are truly pathogenic [43]. In Middle Eastern datasets, up to 53% of high-impact coding variants are missing from gnomAD, with similar findings in African cohorts [44, 45]. These gaps are particularly problematic for missense variants, leading to persistently high VUS rates [42, 46, 47]. Most variants and P/LP assertions are concentrated in a few major European populations [48]. Self-reported race or ethnicity does not accurately reflect genetic ancestry, and ancestry-specific allele frequencies are most reliable in large, well-characterized cohorts [42, 49, 50]. Our focus on ancestry-aware evidence and a strict PM2-supporting definition aims to improve equity while maintaining a conservative approach. As resources such as gnomAD, UK Biobank, regional initiatives like the Qatar Genome Program, and disease- and ancestry-aware thresholds expand, allele frequency priors should stabilize, and VUS burdens are expected to decline [32, 51–53].

### Missense downgrades vs splice-altering upgrades

The differing patterns for missense and splice-altering variants highlight how ACMG/AMP evidence categories function distinctly in *BRCA1*. Missense variant downgrades were primarily driven by calibrated population-frequency data. In contrast, splice-altering variants required transcript-aware PVS1 assessment and rarely met criteria for pathogenic or likely pathogenic classification. This asymmetry also reflects the dataset composition, which was predominantly missense variants (97.1%) and included few splice and nonsense variants. In contrast to the nine missense variants downgraded based on accumulated benign evidence, only one splice-altering variant was upgraded to likely pathogenic through the combined application of PVS1 and PM2. This asymmetry underscores the distinct evidentiary patterns between the two variant classes: ancestry-aware population data primarily enabled benign classification of missense VUS, while pathogenic upgrades among splice-altering variants were rare and required both loss-of-function and rarity evidence. Overall, these results demonstrate that downgrading occurred more frequently than upgrading in this *BRCA1* VUS dataset.

These divergent patterns reflect differences in how ACMG/AMP criteria apply to *BRCA1*’s mechanism [54]. For splice-altering variants, rigorous PVS1 assessment requires confirming loss of function as the relevant mechanism, considering gene position, and distinguishing exon-skipping events that may preserve function [53, 55]. This process is particularly complex in *BRCA1/2*, where extensive alternative splicing and functional isoforms necessitate complementary assays such as RT-PCR and RNA massively parallel sequencing (RNA-MPS) [56].

Current frameworks incorporate specialized splicing codes (such as PVS1_O and BP7_O) with graded strengths and rules for multi-transcript readouts that consider coding potential and relative expression [36]. Combined with calibrated functional data, these approaches support variant-specific probabilistic assessments using likelihood-ratio methods, which can outperform traditional odds-ratio approaches and enable more nuanced evidence weighting [57]. In our dataset, these principles primarily resulted in conservative upgrades for a minority of splice-altering variants and benign-leaning reclassification of missense variants based on allele-frequency data.

### Functional evidence: Status and future integration

Our primary signals were derived from population data and predicted loss-of-function/splicing evidence [42, 58], but high-throughput functional assays provide a critical orthogonal layer for future reinterpretation. Saturation genome editing in *BRCA1* and *BRCA2* has demonstrated high classification accuracy for distinguishing P/LP from Benign/Likely Benign (B/LB) variants [59, 60]. In parallel, deep-learning models such as EVE can perform comparably to high-throughput experiments for genes with rich clinical data, suggesting that experimental efforts may be most impactful where computational predictions are weakest [61].

As laboratory standards for PS3/BS3 mature, validated functional assays are likely to resolve borderline VUS, particularly missense variants in constrained domains [58, 60]. New platforms that couple variant perturbations to complex cellular phenotypes in primary human cells further extend this capacity, though they still face limitations in sensitivity and coverage of gene expression contexts [62]. Integrating these data under standardized validation criteria should harmonize classifications across laboratories and complement ancestry-aware population evidence [58, 63].

### Clinical relevance and counseling implications

Reducing the rate of VUS yields direct clinical benefits for genetic counseling, cascade testing, and patient management by preventing unnecessary interventions for benign-leaning variants and facilitating appropriate surveillance and risk reduction for credible LP/P findings [34]. Ancestry-informed allele-frequency thresholds are especially critical in underrepresented populations, where false rarity and missed downgrades may perpetuate health disparities. While VUS should not inform clinical care, they often generate uncertainty, anxiety, and misinterpretation, which can result in unwarranted procedures. This highlights the necessity for specialist counseling and scheduled reinterpretation [31, 64]. Reclassification, most often to benign or likely benign, is typically slow and inconsistent [65]. The present findings support the systematic application of ancestry-aware allele frequencies and standardized frameworks to accelerate safe downgrades and prioritize variants for functional follow-up. The clinical relevance of these findings requires cautious interpretation. Reclassification impacted only 10 variants, and no patient-level reassessment or clinical validation was conducted. Therefore, this study demonstrates methodological feasibility and incremental enhancement in evidence calibration rather than direct improvement in patient outcomes. Its primary practical contribution is illustrating how ancestry-aware population data can be transparently integrated into *BRCA1* reinterpretation workflows for underrepresented populations.

Several limitations should be noted. First, the approach primarily relied on population-frequency evidence and calibrated PVS1 assessment, with limited integration of additional ACMG/AMP evidence categories. Second, gnomAD v4 annotated only a subset of variants in the dataset, and the Middle East subset does not adequately represent the full diversity of Arab and neighboring populations. Third, the dataset consisted predominantly of missense variants, which restricts inference for splice-altering and nonsense variant classes. Fourth, the ClinVar categories of VUS and “not provided” are not equivalent; although only one “not provided” variant was present and did not affect reclassification, these labels should not be combined without careful consideration in future studies. Finally, local population databases from Qatar and the broader region would likely offer more informative, ancestry-matched evaluation of rare variants than global reference resources alone.

## Conclusion

An ancestry-aware, variant-type-specific ACMG/AMP framework, implemented in accordance with ENIGMA *BRCA1/2* specifications and supported by a points-based system, provided a feasible and auditable approach for *BRCA1* VUS reinterpretation. Ancestry-stratified allele-frequency data yielded a modest but measurable improvement over global-only analysis, contributing to nine missense downgrades, including five supported by the Middle East subgroup and others supported by additional gnomAD ancestry strata. The framework also supported upgrading one splice-altering variant to likely pathogenic based on combined PVS1 and PM2 supporting evidence, demonstrating that variant-type-specific interpretation can inform both benign and pathogenic outcomes. However, most variants remained unresolved, indicating that population data alone are insufficient and should be integrated with larger regional reference datasets, functional studies, and case-based evidence to support more definitive clinical interpretation.

## Supplemental data

**1. Supplemental Data 1 (Excel): Filtered ClinVar dataset**

Filtered ClinVar dataset of 626 BRCA1 variants, including missense, splice-altering, and nonsense variants annotated as VUS or “not provided.” The file contains genomic coordinates, consequence terms, review status, and submitter details. Available at: https://www.protocols.io/file/v2xhcu347.xlsx.

**2. Supplemental Data 2 (Text): Intermediate variant table**

Eight-column table (CHR, POS, ID, REF, ALT, QUAL, FILTER, INFO) used to generate the study VCF file (list_variants.vcf). Available at: https://www.protocols.io/file/v2xecu347.txt.

**3. Supplemental Data 3a–b (VCF and index): Study VCF prior to annotation**

Bgzip-compressed study VCF (list_variants.vcf.gz) and corresponding tabix index file (.tbi) containing all 626 BRCA1 variants prior to annotation.

Supplementary Data 3a: https://www.protocols.io/file/v2xacu347.gz

Supplementary Data 3b: https://www.protocols.io/file/v2xccu347.tbi

**4. Supplemental Data 4a–b (Annotated VCF and index): gnomAD v4 annotation**

Bgzip-compressed VCF file and corresponding tabix index generated after annotation with gnomAD v4 genome and exome allele frequencies for all variants.

Supplementary Data 4a: https://www.protocols.io/file/v2w9cu347.gz

Supplementary Data 4b: https://www.protocols.io/file/v2xfcu347.tbi

**5. Supplemental Data 5a–b (Annotated VCF and index): dbNSFP v4.6 annotation**

Bgzip-compressed VCF file and corresponding tabix index generated after annotation with dbNSFP v4.6 in silico prediction scores, including REVEL, CADD, MetaSVM, MetaLR, MetaRNN, DANN, SIFT4G, and FATHMM.

Supplementary Data 5a: http://www.protocols.io/file/v2xbcu347.gz

Supplementary Data 5b: https://www.protocols.io/file/v2xgcu347.tbi

**6. Supplemental Data 6 (Text): Simplified analytical dataset**

Simplified tab-delimited dataset extracted from the fully annotated VCF, retaining only fields required for ACMG/AMP evidence assignment and subsequent analyses. Available at: https://www.protocols.io/file/v2xdcu347.txt

**7. Supplemental Data 7a-d (Excel): Final analytical workbook**

Final analytical workbook integrating all annotations, including gnomAD v4 and dbNSFP v4.6, with assigned ACMG/AMP evidence codes for each of the 626 BRCA1 variants. This file underpins all reclassification results reported in the manuscript. Available at: https://www.protocols.io/file/v2xicu347.xlsx

**8. Supplemental Data 8: Reproducible workflow**

A fully reproducible workflow, including all commands and configuration files used at each analysis step, is available at:https://www.protocols.io/blind/8D471763C78311F091A60A58A9FEAC02

## Data Availability

The data that supports the findings of this study are available in the supplementary material of this article. Additionally, a fully reproducible workflow, including all commands and configuration files used in each step, is available at: https://www.protocols.io/blind/8D471763C78311F091A60A58A9FEAC02
